# Predicting the diagnosis of prostate cancer with a scoring system based on novel biomarkers

**DOI:** 10.1186/s12894-022-00956-2

**Published:** 2022-02-02

**Authors:** Durvesh Lachman Jethwani, Lameena Lalitha Sivamoorthy, Charng Chee Toh, Rohan Malek

**Affiliations:** 1 Department of Urology, Hospital Selayang, Batu Caves, Selangor Malaysia; 2 Department of General Surgery, Hospital Selayang, Batu Caves, Selangor Malaysia

**Keywords:** Prostate cancer, Novel biomarker ratios, Neutrophil-to-lymphocyte ratio, PSA density, TRUS biopsy

## Abstract

**Objective:**

To predict prostate cancer using novel biomarker ratios and create a predictive scoring system.

**Materials and methods:**

Data of a total of 703 patients who consulted Urology Department of Selayang Hospital between January 2013 and December 2017 and underwent prostate biopsy were screened retrospectively. Prostate specific antigen (PSA) levels, prostate volumes (PV), neutrophil and lymphocyte counts, neutrophil-to-lymphocyte ratio (NLR), Prostate specific antigen density (PSAD) and histopathology were evaluated.

**Results:**

Ages ranged from 43 to 89 years, divided into 2 groups as per biopsy results; positive for prostate cancer (n = 290, 41.3%) and negative for malignancy (n = 413; 58.7%). Intergroup comparative evaluations were performed. Independent variables with *p* < 0.001 in the univariate analysis were age, DRE, PV, NLR, PSAD. A scoring system was modelled using NLR < 0.9, PSAD > 0.4, Age > 70 and DRE. A score of 2 or more predicted prostate cancer with a Sensitivity of 83.8% and Specificity of 86.4%.

**Conclusions:**

NLR is shown to be good predictor for prostate cancer its usage in this scoring system affords more disease specificity as compared to PSA alone.

## Background

Prostate cancer is the fifth most common cancer amongst the male population in Malaysia, with a lifetime risk of developing cancer being 1 in 117 [1]. Transrectal ultrasound guided biopsies (TRUS) remains the gold standard for diagnosing prostate cancer, though not without its own set of complications. The list of complications includes per rectal bleeding, hematuria, and sepsis. Prostate specific antigen (PSA) is a polypeptide that is expressed at very high levels in prostate epithelial cells that was first discovered in 1988. It has since been used widely as a screening tool for prostate cancer. However, it lacks in disease specificity and can be raised in various other conditions including prostate inflammation and benign prostatic hyperplasia. This has led to many unnecessary biopsies. Hence, there is a necessity to develop other screening tools. There have been various studies comparing other markers including PSA derivatives and inflammatory parameters as an additional screening tool. In recent years, the role of inflammation in carcinogenesis and aggressiveness of the cancer has been studied in various types of cancer. A tissue microenvironment is created by inflammation via increased cell replication, angiogenesis, and tissue repair, which are all related to carcinogenesis. There has been more emphasis on inflammatory markers as a tool in not only diagnosing but also as a prognostic indicator for many cancers including lung, colorectal, pancreatic, ovarian, and prostate cancer. These markers include C-reactive protein (CRP), platelet counts, neutrophil counts, neutrophil-lymphocyte ratio (NLR), and platelet-lymphocyte ratio [2–6]. The rationale of this study is to improve the accuracy in detecting prostate cancer by using novel biomarker ratios. NLR, PV & PSAD have not been used together for prediction of prostate cancer in previous studies. If validated, it may be a useful and inexpensive tool in predicting prostate cancer, thus reducing the number of unnecessary biopsies.

## Objective

### General objective

This study aims to evaluate the ability of novel biomarker ratios in predicting the diagnosis of prostate cancer.

### Specific objectives

1. To determine the association between novel biomarker ratio (NLR to PV & neutrophil count to PV & NLR to PSAD) and prostate cancer
2. To determine the area under the curve, cut off values, sensitivity, and specificity for the above in predicting prostate cancer
3. To develop a scoring system between these biomarkers and other factors (e.g., age and abnormal DRE) to predict the diagnosis of prostate cancer

## Material and methodology

**Fig. 1:**
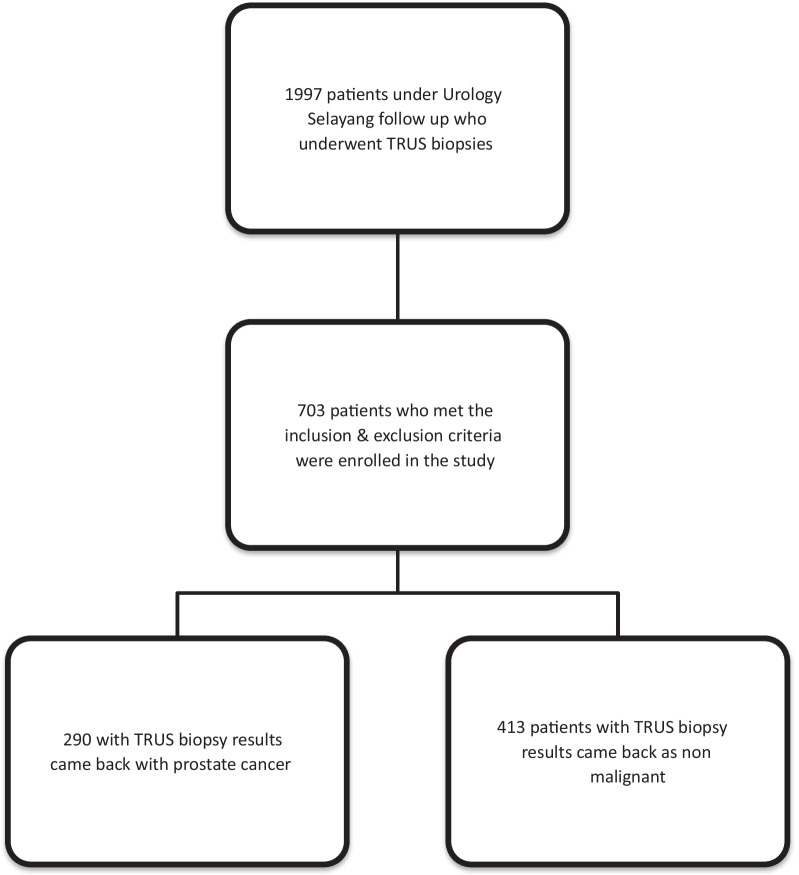
Methodology flowchart

This single center retrospective case-control study was approved by our institutional ethics review board and Medical Research Ethics Committee. All patients who underwent a TRUS biopsy from January 2013 to December 2017 were recruited. All data was analyzed anonymously. All men underwent TRUS prostate biopsies secondary to elevated PSA levels or abnormal digital rectal exams (DRE). In all men, the prostate was routinely biopsied bilaterally near the base, mid-gland region, and apex, taking at least six biopsies per side. The control group were those whose biopsies turned out negative for malignancy. Inclusion criteria were all patients who underwent a TRUS biopsy during the study duration and had a full blood count result within 6 months of the biopsy. Excluded from study were those who were previously diagnosed with prostate cancer, had previous prostate surgery as it may interfere to inaccurate volume calculation, previous or on-going treatment with 5-α reductase inhibitor (5-ARI), those with Full Blood Count (FBC) taken during an acute illness (e.g., respiratory tract infection, fever), those who were on immunosuppressants such as steroids, and PSA taken while on catheter or suffering from prostatitis. Out of 1997 patients that underwent TRUS biopsies in the stipulated time, 703 patients met the requirements, of which 290 were diagnosed with prostate cancer and 413 had biopsied that returned negative for malignancy. A methodology flowchart is illustrated in Fig. 1.

### Study protocol

Electronic medical records of the study population were examined to retrieve the data needed. Our standard protocol for transrectal prostate biopsy is the 12-quadrant template biopsy with local anesthesia after receiving preoperative administration of antibiotic prophylaxis and distal bowel preparation. Biopsy specimens were evaluated in the same histopathology clinic. PSA levels and prebiopsy whole blood cell counts were obtained from the hospital laboratory. Prostate volume was determined via measurements taken on TRUS. PSA density was calculated by dividing the serum PSA level by the prostate volume on TRUS (maximum longitudinal diameter × maximum transverse diameter × maximum AP diameter × π/6). NLR was calculated as the absolute neutrophil count divided by the absolute lymphocyte count taken from full blood count results.

### Data analysis

Data analysis was performed using SPSS version IBM 23.0. Descriptive analysis was conducted for each variable. A univariate analysis followed by a multivariate regression determined the association between each variable and the novel biomarkers with prostate cancer within the study population. We determined the cut-off point according to the sensitivity and specificity levels derived from area under curve (AUC) for receiver operator characteristics (ROC) curve plotted using the presence or absence of prostatic cancer with Youden Index formula. A scoring system was created using the significant variables. The predictive accuracy (sensitivity & specificity) of the scoring system was assessed using receiver operating characteristic (ROC)-derived area under the curve (AUC) analysis.

## Results

The data of the 703 included patients is displayed in displayed in Table 1. Just over sixty four percent of the patients (186 patients) were diagnosed with clinically significant prostate cancer (Gleason score ≥ 7). The average age (69 ± 7 years) of patients with prostate cancer was older than those without cancer (66 ± 6.5 years). Based on the logistic regression analysis, with an increase in age there was a positive association in developing prostate cancer, with an odds ratio of 1.072 (95% CI 1.047–1.097, *p* < 0.001). Majority of patients were of Malay origin (138 patients with prostate cancer and 233 patients with negative biopsy results). Followed by Chinese ethnicity; accounting for 122 (42.1%) patients with prostate cancer and 146 (35.4%) patients without prostate cancer, then Indians; 27 (9.3%) with prostate cancer and 32 (7.7%) without cancer. This reflected the local demography of the population.

**Table 1:** Characteristics and blood parameters of patients with biopsy results positive for prostate cancer and negative for malignancy

Patient characteristics	Prostate cancer/positive biopsy	Non malignant/negative biopsy	Univariate analysis			Multivariate analysis		
			Odds Ratio	95% Confidence Interval	Significance *p* value	Adjusted Odds Ratio	95% Confidence Interval	Significance *p* value
Number of patients (%)	290 (41.3%)	413 (58.7%)	–	–	–	–	–	–
*PCa (%)*								
Gleason ?3 + 4 (ISUP 1, 2)	104 (35.9%)	–	–	–	–	–	–	–
Gleason ?4 + 3 (ISUP 3,4,5)		–						
	186 (64.1%)							
*Age (years)*			1.072	1.047–1.097	< 0.001	1.093	1.054–1.134	< 0.001
Mean (± SD)	69 (± 7.0)	66 (± 6.5)						
Median	70	66						
Range	43–86	46–89						
*Ethnicity (%)*								
Malay	138 (47.6)	233 (56.4)	1		0.127			
Chinese	122 (42.1)	146 (35.4)	0.395	0.065–2.392	0.312	–	–	–
Indian	27 (9.3)	32 (7.7)	0.557	0.092–3.388	0.525			
Others	3 (1.0)	2 (0.5)	0.563	0.087–3.617	0.545			
*Hypertension*								
No	145 (50.0)	221 (53.5)	1			–	–	–
Yes	145 (50.0)	192 (46.5)	1.151	0.852–1.555	0.359			
*Diabetes*								
No	215 (74.1)	325 (78.7)	1			–	–	–
Yes	75 (25.9)	88 (21.3)	1.288	0.905–1.834	0.159			
*Chronic kidney disease*								
No	281 (96.9)	393 (95.2)	1			–	–	–
Yes	9 (3.1)	20 (4.8)	0.629	0.282–1.403	0.257			
*Heart disease*								–
No	260 (89.7)	371 (89.8)	1			–	–	
Yes	30 (10.3)	42 (10.2)	1.019	0.622–1.671	0.94			
DRE (%)								
Normal	177 (61.0%)	304 (73.6%)	1					
Abnormal	113 (39.0%)	109 (26.4%)	1.562	1.407–1.775	< 0.001	0.848	0.511–1.408	0.525
***Prostate volume/PV (g)***			0.986	0.980–0.992	< 0.001	0.971	0.962–9.982	< 0.001
Mean (± SD)	46.8 (± 30.4)	57.8 (± 29.4)						
Median	40	51.3						
Range	10.0–287.0	10.0–200.0						
*Neutrophil count (*× *10*^*9*^*)*			0.996	0.979–1.013	0.616			
Mean (± SD)	8.04 (± 8.57)	8.38 (± 9.36)						
Median	5	5				–	–	–
Range	0.40–50.60	1.60–87.10						
*Lymphocyte count (*× *10*^*9*^*)*			9.249	5.289–16.175	< 0.001			
Mean	22.86 (11.2)	2.01 (0.72)						
Median	24.43	1.9				–	–	–
Range	1.90–54.35	0.30–5.10						
***NLR***			0.61	0.543–0.684	< 0.001			
Mean (± SD)	1.15 (± 2.80)	4.91 (± 6.47)						
Median	0.2	2.53				–	–	–
Range	0.03–20.67	0.79–87.1						
***PSA (ng/ml)***			1.051	1.039–1.063	< 0.001	–	–	–
Mean (± SD)	238.15 (± 640.98)	11.93 (± 14.00)						
Median	47.18	8.6						
Range	0.06–5877.99	0.34–191.61						
***PSAD (ng/ml/g)***			5.977	3.953–9.036	< 0.001			
Mean (± SD)	6.48 (± 21.89)	0.27 (± 1.08)						
Median	1.05	0.17				–	–	–
Range	0.01–273.90	0.01–21.74						
***NLR:PV***			1E − 04	0.000–0.002	< 0.001			
Mean (± SD)	0.035 (± 0.109)	0.115 (± 0.207)						
Median	0.006	0.052				–	–	–
Range	0.001–1.254	0.005–2.524						
***Neut:PV***			1.483	0.920–2.392	0.106			
Mean (± SD)	0.235 (± 0.341)	0.194 (± 0.312)						
Median	0.133	0.1				–	–	–
Range	0.016–2.885	0.015–4.291						
***NLR:PSAD***			0.977	0.968–0.985	< 0.001	1.004	1.002–1.006	< 0.001
Mean (± SD)	10.03 (± 68.96)	42.19 (± 112.21)						
Median	0.22	16.94						
Range	0.001–754.55	0.21–1907.30						

Approximately 50–53.5% of patients had hypertension, 20–25% had diabetes, 3.1–4.8% had chronic kidney disease and 10% had heart disease. None of these illnesses appear to influence the rate of prostate cancer. Patients with abnormal digital rectal examination (DRE) were 56.2% more likely to have prostate cancer (*p* < 0.001). Prostate volumes were smaller in the prostate cancer arm 46.8±30.4 mls as opposed to the control arm 57.8 ± 29.4 mls (*p* < 0.001).

**Fig. 2:**
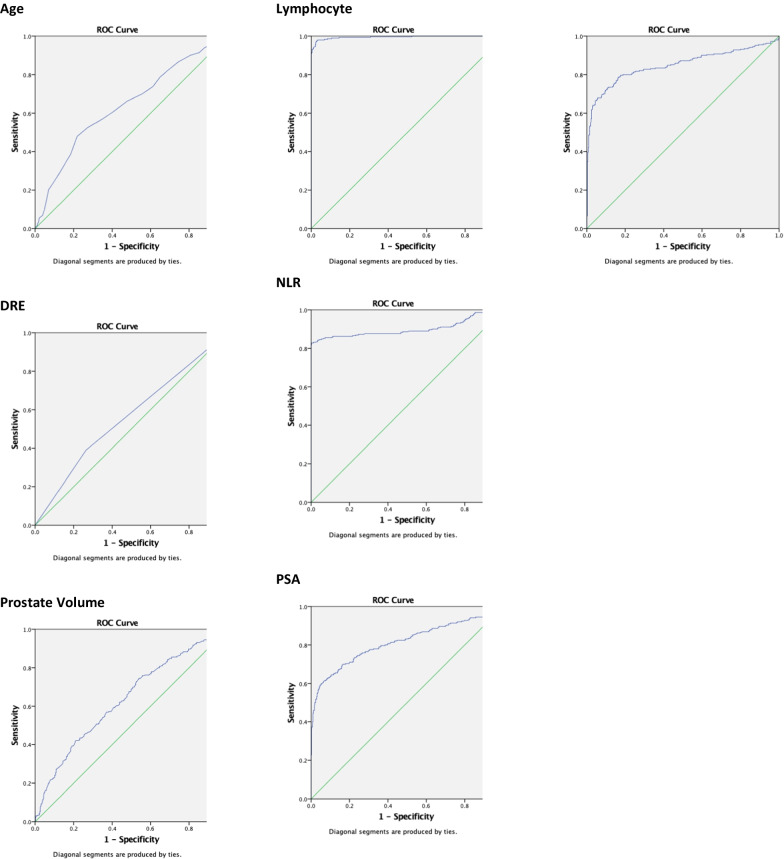
ROC curves for significant variables

Next, we conducted univariate analysis of all the blood parameters and biomarker ratios, and neutrophil count was comparable between both groups. However, both lymphocyte and NLR showed a significant difference between the two groups. Both PSA and PSAD were higher in the prostate cancer arm; mean 238.15 (± 640.98) and 6.48 (± 21.89) respectively versus 11.93 (± 14.00) and 0.27 (± 1.08). NLR: PV and NLR: PSAD were lower in prostate cancer; 0.035 (± 0.109) versus 0.115 (± 0.207) and 10.03 (± 68.96) versus 42.19 (± 112.21) respectively (*p*<0.001). The plotted curves are seen in Fig. 2.

**Fig. 3:**
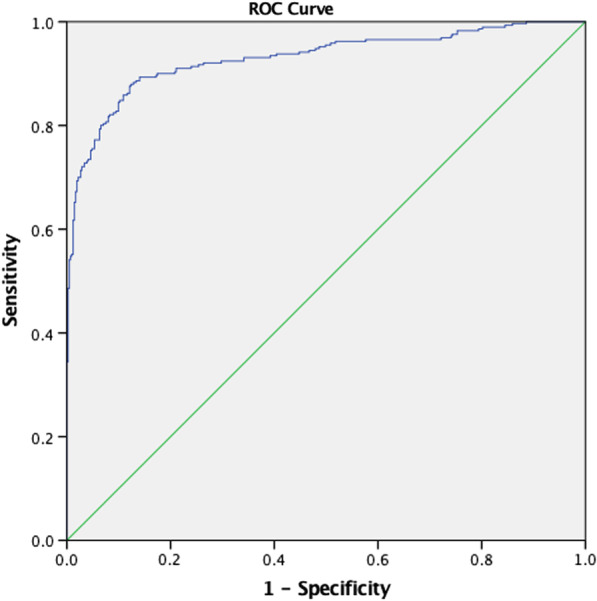
ROC curve for multivariate regression

Independent variables with *p* < 0.001 in the univariate analysis (age, DRE, PV & NLR: PSAD) were selected for multivariate logistic regression. Lymphocyte count, PSA, PSAD & NLR: PV were excluded for multicollinearity issue. A ROC curve as seen in Fig. 3 was plotted using the predictive probability. The ROC curve generated had an AUC of 0.903 (*p* < 0.001, 95% CI 0.909–0.950) with sensitivity of 87.9% and 87.7%.

**Fig. 4:**
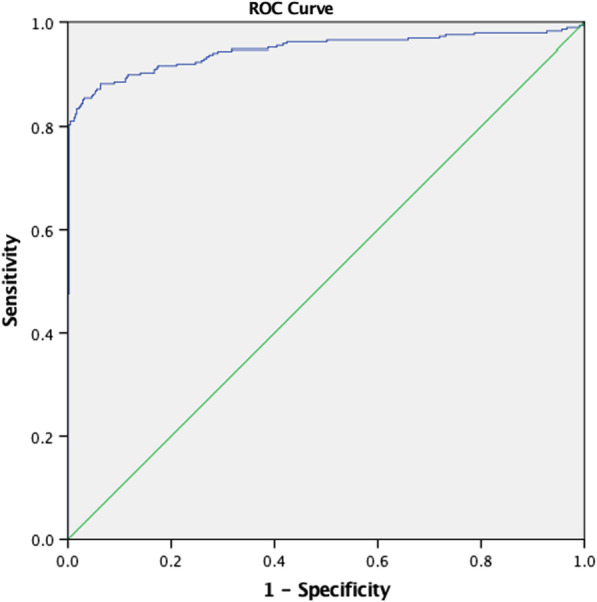
ROC curve for NLR:PSAD

Following which we conducted ROC curve analysis for the significant clinical parameters and biomarkers as per Table 2. The AUC for NLR was 0.901 with cut off value 0.904 had a sensitivity of 82.8% and specificity of 99.8 (*p* < 0.001). PSA and PSAD had near comparable values with AUC 0.813 and 0.849 respectively with low sensitivity 60.0% and 73.1% but high specificity of 94.7% and 89.3%. As seen in Fig. 4, NLR: PSAD was a significant biomarker ratio with AUC of 0.946, sensitivity of 85.5%, specificity of 96.9%.

**Table 2:** ROC curve analysis of variables

	AUC	Cut off	Sensitivity Sn	Specificity Sp	Youden Index	95% Confidence Interval	Significance *p* value
Age	0.640	70.5	47.9	78.2	0.261	0.598–0.682	< 0.001
DRE	0.563	–	39.0	73.6	–	0.520–0.600	0.005
Prostate volume	0.638	35.35	42.1	78.9	0.21	0.596–0.680	< 0.001
Lymphocyte	0.995	3.50	97.6	97.1	0.947	0.990–0.999	< 0.001
NLR	0.901	0.904	82.8	99.8	0.825	0.871–0.930	< 0.001
PSA	0.813	27.54	60.0	94.7	0.577	0.778–0.849	< 0.001
PSAD	0.849	0.409	73.1	89.3	0.624	0.815–0.882	< 0.001
Neut: PV	0.579	0.108	63.4	54.3	0.182	0.536–0.621	< 0.001
NLR: PV	0.881	0.126	75.5	98.5	0.741	0.850–0.913	< 0.001
NLR: PSAD	0.946	3.303	85.5	96.9	0.824	0.926–0.967	< 0.001
Multivariate regression	0.930	–	87.9	87.7	–	0.909–0.950	< 0.001
Scoring system	0.915	2	83.8	86.4	0.702	0.895–0.936	< 0.001

**Fig. 5:**
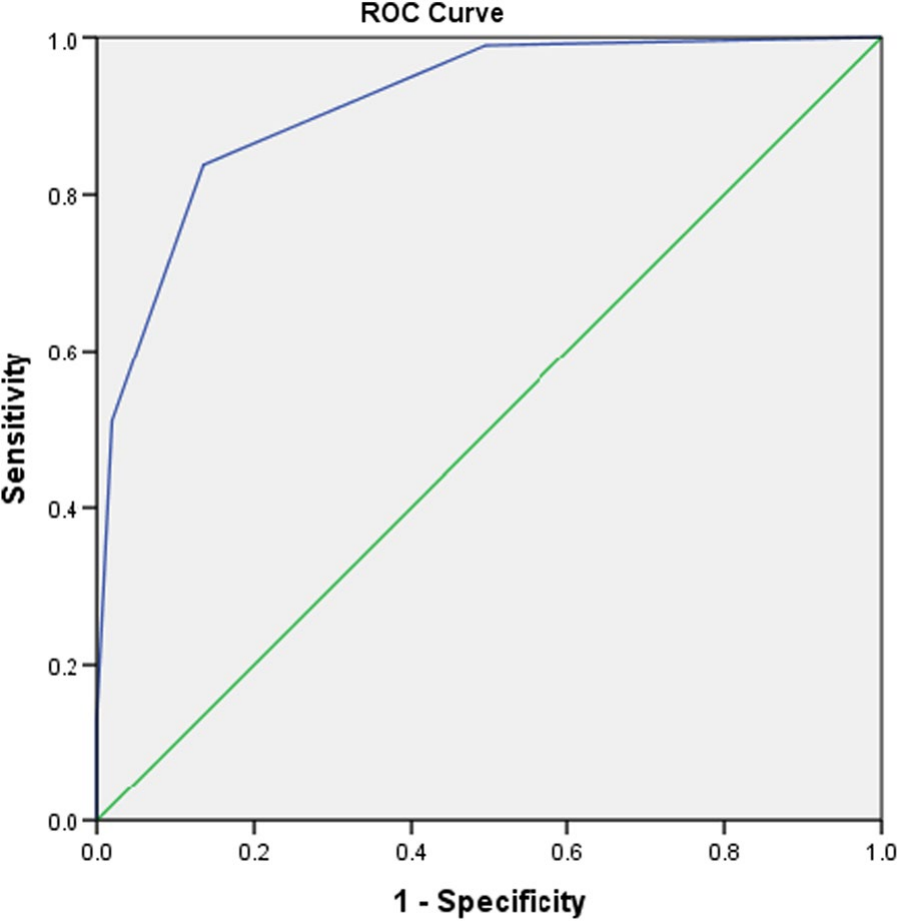
ROC curve for scoring system

A scoring system to predict the probability of having prostate cancer among patients was proposed, as seen in Table 3. It is composed of 4 clinical parameters: age, abnormal DRE, NLR and PSAD. A cut off value of ≥ 2, places the patient at a higher risk of having prostate cancer. The generated ROC curve is seen in Fig. 5. By using the score of 2 as a cut off value, the model had a sensitivity of 83.8% and specificity of 86.4% in predicting prostate cancer.

**Table 3:** Scoring system

Clinical factors	Score
Age > 70	1
Abnormal DRE	1
NLR < 0.9	1
PSAD > 0.4	1
Total	4

## Discussion

Most prostate cancer patients are asymptomatic as it is indolent unlike other cancers. Those with low-risk disease as per the D’Amico criteria, may not require radical therapy. Therefore, there has been a shift in the treatment paradigm, from diagnosing all cancers, to differentiating clinically significant prostate cancer to prevent over treatment or under treatment of the disease.

In our study we created a scoring system that was designed to be simple and easily applicable in the clinic setting. There are only 4 components: age, abnormal DRE findings, NLR and the PSAD. An ROC curve was plot and yielded promising results, with an AUC for clinically significant cancer (GS ≥ 7) of 91.5% (95% CI 0.89–0.93). From this ROC curve, we determined that patients with a score of 2, likely had clinically significant prostate cancer with a sensitivity of 83.8% and specificity of 86.4%.

Two of the more popular scoring systems that have been validated are the Prostate Cancer Prevention Trial Risk Calculator 2.0 (PCPT RC) and the newer Prostate Biopsy Collaborative Group (PBCG) Risk Calculator. Ankerst et al. derived a median AUC of 74.4% (range 62.1–88.1) when designing the Second version of the (PCPT RC) [7]. Similarly, the PBCG calculator achieved an AUC of 75.5% (95% confidence interval 74.2–76.8) in their cohorts [8].

One of the key features of our scoring system was the inclusion of NLR, a novel biomarker. Toriola et al., Kawahara et al. and Oh et al. reported that pre-diagnostic inflammatory markers had a significant positive association with prostate cancer [9–11]. NLR has been shown to be associated with more aggressive disease and higher GS [12, 13]. In our study we found at a cut off less than 0.9, it had an AUC of 90% for clinically significant prostate cancer. NLR is a systemic inflammatory marker that has the advantage of being readily available, convenient, and cost effective.

Carbunaru et al. explored the effectiveness of these calculators in a multi-ethnic cohort and found the AUC for clinically significant Prostate Cancer was lower than the originally published articles at 64% (95% CI 0.61–0.68) for PCPT and 65% (95% CI 0.62–0.68) for PBCG [14]. A limiting factor of using these more established scoring systems, is that they may not reflect the Asian population. In the same study, the authors found that PBCG’s calculator potentially biases a greater number of low-risk African American and other men towards unnecessary biopsies. The need for more data on Asian patients is highlighted by a study by Lim et al. conducted in Malaysia, which found that baseline PSA levels significantly vary amongst different ethnicities [15]. We were able to achieve a good AUC in our study cohort, which fairly reflected the multi-ethnicity of our Malaysian population.

The 2021 edition of the European Association of Urology practice guidelines advocates the use of either risk calculators or imaging (typically in the form of Multiparametric Magnetic Resonance Imaging (MP-MRI)), in asymptomatic men with a PSA < 10 ng/mL [16].

In the PROMIS study, Ahmed et al. found that the usage of a pre-biopsy MP-MRI was highly sensitive (93%) in picking up clinically significant prostate cancer, resulting in the ability to exclude a patient from a biopsy if the result was negative, but with a poor specificity (41%) there were still many patients undergoing biopsies with negative results [17].

To be of good value, MP-MRIs need to adhere to the Prostate Imaging Reporting & Data System (PI-RADS) guidelines for acquisition and interpretation. This requires a significant level of expertise from a trained Radiologist. Paired with the hefty costs of purchasing and maintaining an MRI, there are only a limited number of centers which can cater to timely Pre-biopsy MP-MRIs. Though our scoring system, which excluded the usage of MP-MRIs, resulted in a lower sensitivity, the combination of both Sensitivity and Specificity above 80% makes it a useful tool when counselling a patient about the possible outcomes from a biopsy.

Our study has several limiting factors. One of the main drawbacks of our study is its retrospective nature. Hence, information bias and selection bias could not be avoided. Follow up was not an integral part of our study. We only used data from a single center but we were able to get a good sample size, which represented our local Malaysian population. We also could not use other inflammatory markers such as CRP or procalcitonin to exclude patients with ongoing infection. We acknowledge the need for a prospective validation study to further determine the accuracy of these novel biomarker ratios and scoring system.

Another feature of our scoring system, PSAD, was achieved by measurement of the prostate volume via transrectal ultrasound, which is cumbersome to reproduce, especially if one has not committed to undertaking a biopsy.

## Conclusion

In conclusion, with a higher sensitivity and specificity than DRE or elevated PSA alone, we believe usage of our scoring system could potentially reduce the number of unnecessary TRUS biopsies, which, in turn, will reduce the risk of complications to patients and reduce the burden to the healthcare system. We recommend this scoring system should be used to facilitate the counselling of a patient being considered for a biopsy. However, we acknowledge that prospective studies are needed to validate this.

## Data Availability

The datasets used and/or analyzed during the current study are available from the corresponding author on reasonable request.

## Abbreviations

PSA: Prostate Specific Antigen
PV: Prostate Volume
DRE: Digital Rectal Examination
PSAD: Prostate Specific Antigen Density
NLR: Neutrophil to Lymphocyte Ratio
TRUS: Transrectal Ultrasound
AUC: Area Under Curve
ROC: Receiver Operator Characteristics
CI: Confidence Interval
SD: Standard Deviation
SP: Specificity
SN: Sensitivity
FBC: Full Blood Count
MP-MRI: Multiplanar Magnetic Resonance Imaging
PI-RADS: Prostate Imaging Reporting and Data System
GS: Gleason Score
